# Mild exercise improves executive function with increasing neural efficiency in the prefrontal cortex of older adults

**DOI:** 10.1007/s11357-023-00816-3

**Published:** 2023-06-15

**Authors:** Kyeongho Byun, Kazuki Hyodo, Kazuya Suwabe, Takemune Fukuie, Min-seong Ha, Chorphaka Damrongthai, Ryuta Kuwamizu, Hikaru Koizumi, Michael A. Yassa, Hideaki Soya

**Affiliations:** 1 Sport Neuroscience Division, Advanced Research Initiative for Human High Performance (ARIHHP), Faculty of Health and Sport Sciences, University of Tsukuba, Tsukuba, Ibaraki Japan; 2 Division of Sport Science; Sport Science Institute & Health Promotion Center, College of Arts & Physical Education, Incheon National University, Yeonsu, Incheon Republic of Korea; 3 Physical Fitness Research Institute, Meiji Yasuda Life Foundation of Health and Welfare, Hachioji, Tokyo Japan; 4 Laboratory of Exercise Biochemistry and Neuroendocrinology, Faculty of Health and Sport Sciences, University of Tsukuba, Tsukuba, Ibaraki Japan; 5 Faculty of Health and Sport Sciences, Ryutsu Keizai University, Ryugasaki, Ibaraki Japan; 6 Department of Sports Science, College of the Arts and Sports, University of Seoul, Dongdaemun, Seoul Republic of Korea; 7 Department of Neurobiology and Behavior, Center for the Neurobiology of Learning and Memory, University of California, Irvine, CA USA

**Keywords:** Mild-exercise intervention, Executive function, Neural efficiency, Functional near-infrared spectroscopy, Prefrontal cortex

## Abstract

This study examined whether a 3-month mild-exercise intervention could improve executive function in healthy middle-aged and older adults in a randomized control trial. Ultimately, a total of 81 middle-aged and older adults were randomly assigned to either an exercise group or a control group. The exercise group received 3 months of mild cycle exercise intervention (3 sessions/week, 30–50 min/session). The control group was asked to behave as usual for the intervention period. Before and after the intervention, participants did color-word matching Stroop tasks (CWST), and Stroop interference (SI)-related reaction time (RT) was assessed as an indicator of executive function. During the CWST, prefrontal activation was monitored using functional near-infrared spectroscopy (fNIRS). SI-related oxy-Hb changes and SI-related neural efficiency (NE) scores were assessed to examine the underlying neural mechanism of the exercise intervention. Although the mild-exercise intervention significantly decreased SI-related RT, there were no significant effects of exercise intervention on SI-related oxy-Hb changes or SI-related NE scores in prefrontal subregions. Lastly, changes in the effects of mild exercise on NE with advancing age were examined. The 81 participants were divided into two subgroups (younger-aged subgroup [YA], older-aged subgroup [OA], based on median age [68 years.]). Interestingly, SI-related RT significantly decreased, and SI-related NE scores in all ROIs of the prefrontal cortex significantly increased only in the OA subgroup. These results reveal that a long-term intervention of very light-intensity exercise has a positive effect on executive function especially in older adults, possibly by increasing neural efficiency in the prefrontal cortex.

**Supplementary Information:**

The online version contains supplementary material available at 10.1007/s11357-023-00816-3.

## Introduction

Given the expected healthcare cost growth due to the rapid increase in population age in developed countries [1], there has been increasing attention to the potent effects of exercise regimens on age-related cognitive impairment, which is a predictor of independence in old age. Over the past two decades, abundant evidence has shown that physical activity as well as regular exercise confer benefits on brain health and function, particularly in aged populations [2–6]. The majority of past studies have focused on cardiovascular exercise programs expecting them to provide beneficial cognitive effects especially on executive function. Recent neuroimaging studies suggest that exercise-induced efficient neural activity [7, 8] and gray matter volume increases [9–12] in the prefrontal cortex could be potential mediators for exercise’s effects on preventing cognitive impairment in late adulthood.

In addition to a cardiovascular exercise regime, there is also evidence that mild (very light intensity) exercise, which is easily attainable by sedentary people and those with low aerobic fitness without eliciting stress responses, has positive effects on brain structure and function. A longitudinal exercise intervention study performed with older adults demonstrated that 2 years of mild-intensity calisthenics was effective in improving executive function, which was positively correlated with morphological changes in the prefrontal cortex [13]. In addition to morphological changes, acute mild exercise can elicit increasing task-related brain activations resulting in better cognitive functions in young adults [14, 15]. However, it is still unknown whether a regular mild-exercise intervention could prevent age-related cognitive decline in older adults and how regular mild exercise could have an impact on functional neural substrates, particularly in the prefrontal cortex.

Functional near-infrared spectroscopy (fNIRS) can be used to assess cerebrovascular responses in the prefrontal cortex during an executive function challenge such as the color-word matching Stroop task (CWST). It is therefore an appropriate method for examining the potential effects of a chronic, mild-exercise intervention on executive function and its underlying neural mechanisms in older adults. Past work with acute exercise intervention models (mild exercise-, moderate-, and high-intensity intermittent exercise) has shown that an acute bout of exercise temporarily increases activation in task-relevant prefrontal subregions, such as the left dorsal lateral prefrontal cortex (DLPFC) and/or the ventral prefrontal cortex (VLPFC), in young adults, or has been associated with compensatory brain activation in the right frontopolar area (FPA) in older adults, both resulting in better executive performance [14, 16–18]. Based on these findings, we hypothesized that a chronic, mild-exercise intervention would enhance executive function by shortening Stroop interference (SI)-related reaction time (RT) and increasing activation in the lateral prefrontal cortex. We focused on these mechanisms, as they are known to represent the inhibitory control component of executive function.

Functional NIRS coupled with a cognitive task that can manipulate mental workload allows us to measure mental effort correlated to localized hemodynamic changes within task-relevant prefrontal regions [19, 20]. According to the neural efficiency hypothesis [21, 22], cognitive intelligent individuals exhibit less brain activation when performing certain cognitive tasks because such tasks require less mental effort. Studies using the Stroop task have demonstrated that aging is associated with a decline in neural efficiency and neural activity [23] related to conflict processing, which is localized in the prefrontal cortex [24]. In addition, recent functional neuroimaging studies have demonstrated that a chronic exercise intervention leads to improved cognitive function without substantially increased task-related neural resources to deal with conflict processing after exercise intervention [25–27]. Hence, combining fNIRS and Stroop interference provides an opportunity to test the hypothesis that a regular, mild-exercise intervention improves executive function by changing neural efficiency in the prefrontal cortex.

Importantly, since both executive performance and recruitment of task-relevant neural resources might be differentially affected by advancing age [24, 28–30], neural efficiency in dealing with SI-elicited cognitive conflicts could deteriorate with age, particularly in the prefrontal cortex. Moreover, cardiovascular fitness levels, which can be maintained or improved with regular exercise, are related to changes in cognitive function in younger older-adults and older older-adults in different ways [31, 32]. Specifically, Goenarjo and colleagues revealed that high-fitness older older-adults exhibit better executive performance with higher oxygenation in the prefrontal cortex, but that younger older-adults do not [32]. Thus, it is possible to postulate that the neural basis for regular mild exercise effects on human executive function would differ with advancing age. This could especially be the case from the viewpoint of neural efficiency [33, 34].

In the present study, we first examined whether a 3-month mild-exercise intervention can improve executive function in older adults. Then, using a multichannel fNIRS to monitor changes in lateral prefrontal cortex activation in response to SI, we further investigated how this mild-exercise intervention affects neural substrates in the prefrontal cortex in older adults, based on the concept of neural efficiency. We hypothesized that a long-term mild-exercise intervention will enhance executive performance by shortening RT in response to SI, which would in turn be related to increases in neural activations, as with acute exercise models, or enhanced neural efficiency of the prefrontal cortex in older adults. Lastly, we examined whether the positive impact of a mild-exercise intervention on neural efficiency would be more evident in a relatively older age group.

## Methods

### Participants

Community-dwelling healthy older adults (*n* = 125) were recruited to the intervention. One hundred and ten participants were enrolled in the study after a telephone screening regarding their regular exercise habits and medical history as well as other eligibility criteria. All eligible participants were right-handed Japanese native speakers, were between the ages of 55 and 80 years, had no history of psychiatric or neurological disorders, were not undergoing hormone therapy, had normal or corrected-to-normal vision with normal color vision, had no history of cardiovascular disease or diabetes, were naïve to the experimental procedures for which they had volunteered, had not committed to any regular exercise in the previous 6 months, and signed an informed consent form approved by the University of Tsukuba. The trial registration number is UMIN000049392.

**Fig. 1:**
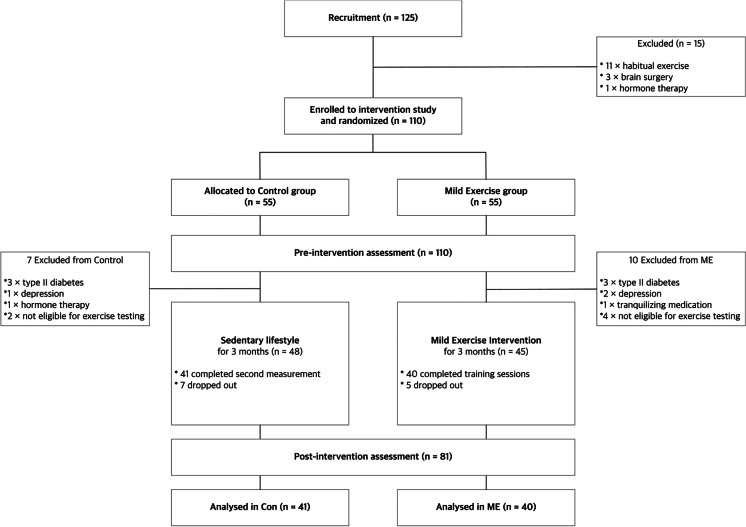
Flow of 3-month intervention study

Demographic information is found in Table 1. After excluding 15 potential participants for non-eligibility (exercise habit [*n* = 11], brain surgery [*n* = 3], hormone therapy [*n* = 1]) during initial screening, the remaining 110 participants were randomly assigned to either a mild exercise group (ME; *n* = 55) or a control group (Con; *n* = 55), which were matched based on age and sex. After baseline measurements, the following participants were also excluded: 6 participants (3 Con and 3 ME) with type II diabetes mellitus; 3 participants (1 Con and 2 ME) with depressive symptoms over 10 on the Geriatric Depression Scale 15-item short form; 1 participant undergoing hormone therapy (1 Con); 1 participant on tranquilizing medication (1 ME); and 6 participants (2 Con and 4 ME) because they were not able to complete the aerobic fitness test using a recumbent cycle ergometer and a gas analyzer. Additionally, 5 participants in Con and 7 participants in ME did not complete the intervention. Ultimately, 81 participants (73.6% of participants initially enrolled; 41 Con and 40 ME) remained and were included in the analyses (Fig. 1). We determined the prospective power with this sample size using G-power software [35] based on our previous acute exercise intervention studies [14, 17], and 81 participants were considered sufficient to detect a significant difference between groups (Con vs ME) with a two-sided, alpha of 0.05 on a test of proportions (difference between two dependent means [matched pairs]) with 95% power.

**Table 1:** Participant characteristics

Variables	All participants *n* = 81, 20 males	ME group *n* = 40, 10 males	Control group *n* = 41, 10 males	*p*-value
Age (years)	68.2 (67.0–69.4)	68.7 (67.0–70.3)	67.7 (65.9–69.6)	0.449
Height (cm)	156.6 (154.9–158.3)	156.5 (153.9–159.1)	156.8 (154.4–159.1)	0.871
Weight (kg)	56.0 (53.5–58.5)	55.6 (52.4–58.9)	56.4 (52.5–60.3)	0.776
${\dot{\mathrm V}}_{{\mathrm O}_2\mathrm{peak}}$ (ml・min・kg^-1^)	23.0 (22.0–23.9)	23.2 (21.9–24.6)	22.7 (21.3–24.1)	0.598
Education (years)	13.6 (13.2–14.1)	13.4 (12.7–14.1)	13.9 (13.3–14.5)	0.346
MMSE (score)	29.0 (28.8–29.3)	29.1 (28.7–29.4)	29.0 (28.6–29.4)	0.849

All values are presented as mean with 95% confidence interval. Note. *ME*, mild exercise; *Con*, Control; ${\dot V}_{O_2peak}$, peak oxygen consumption; *MMSE*, Mini-Mental Status Examination

### Cardiovascular fitness-related variables

Maximal oxygen uptake (${\dot{\mathrm V}}_{{\mathrm O}_2\mathrm{peak}}$) was used to assess baseline cardiovascular fitness levels and to determine the appropriate individual intensity for mild exercise adopted in the mild-exercise intervention program. Participants were required to obtain consent from their physician before fitness tests were conducted. Aerobic fitness was assessed using a ramp protocol until voluntary exhaustion with an electronically braked cycle recumbent ergometer (Corival Recumbent Cpet, Lode, Netherlands) and a gas analyzer (AE310s, Minato Medical Science, Japan) to measure breath-by-breath oxygen intake (${\dot{\mathrm V}}_{{\mathrm O}_2}$) and carbon dioxide output ($\dot{\mathrm V}_{{\mathrm C}{\text{O}}_2}$) at a sampling rate of 0.1 Hz. Participants began cycling for 2 min at 0 W, followed by a gradual increase in power demanded at a rate of 10 W per min. During the cardiovascular fitness test, each participant’s heart rate and respiratory parameters were recorded, and the level of Borg’s rating of perceived exertion (RPE) was measured at the 45th second of every minute [36]. ${\dot{\mathrm V}}_{{\mathrm O}_2\mathrm{peak}}$ was determined once two of the following criteria were satisfied: the respiratory exchange ratio (${\dot{\mathrm V}}_{{\mathrm O}_2}$ / $\dot{\mathrm V}_{{\mathrm C}{\text{O}}_2}$ ratio) exceeded 1.0, 90% of the age-adjusted estimated maximal heart rate was reached, or an RPE of 19 or 20 was reached [37–39].

### Mild-exercise intervention

Participants assigned to the ME group participated in three exercise sessions per week for 12 weeks, supervised by qualified exercise specialists. For safety, participants self-monitored their resting heart rate and blood pressure and reported them, along with any daily subjective symptoms they may have been experiencing, to trainers before every session. Participants started by cycling for 30 min, and the duration was increased monthly by 10-min increments until a duration of 50 min was achieved at month 3. All cycling sessions started and ended with 5 min for warming up and cooling down. Trainers adjusted the individual workload of the recumbent ergometer (Monark RT2, Vansbro, Sweden) to correspond to 35% of the ${\dot{\mathrm V}}_{{\mathrm O}_2\mathrm{peak}}$ of each participant as mild exercise (very light-intensity exercise is defined as under 37% of a subject’s ${\dot{\mathrm V}}_{{\mathrm O}_2\mathrm{peak}}$ by the classification of physical activity intensity of the American College of Sports Medicine [40]. Heart rate was recorded at the beginning and end of each session using a heart rate monitor (Polar OH1, Kempele, Finland) worn on the participant’s forearm. Exercise program attendance was recorded by trainers, and each participant’s attendance level was calculated as the number of sessions attended divided by the total number of exercise sessions. Participants in the Con group were required to maintain their normal, daily physical activity level.

### Behavioral measurements

The color-word matching Stroop test, as used in event-related designs in previous studies [14, 17], was modified to evaluate the executive performance of healthy older adults. It consisted of thirty trials, involving ten neutral, ten congruent, and ten incongruent trials presented in random order. For the neutral trials, the upper row contained a set of X’s (XXXX) presented in blue, green, red, or yellow, and the lower row contained the words “BLUE,” “GREEN,” “RED,” or “YELLOW” presented in black. For congruent trials, the upper row consisted of the words “BLUE,” “GREEN,” “RED,” or “YELLOW” presented in a congruent color (e.g., BLUE presented in blue). For incongruent trials, the color word in the upper row was presented in an incongruent color (e.g., BLUE presented in red), and the lower row contained color words presented in black to produce cognitive interference between the color of the word and the color’s name.

Participants were instructed to determine whether the color of the upper letters matched the meaning of the lower letters. The lower letters were presented 350 ms after the upper letters to obtain sequential visual attention. Participants needed to press a “yes” or “no” button with their index fingers to respond. The stimulus remained on the screen until the response was made or for a maximum of 3 s. Trials were separated with an inter-stimulus interval containing a fixation cross mark for a variable period between 10 and 12 s to prevent anticipation of the timing of the onset of the next stimulus. The left–right order of the two buttons was randomly counterbalanced across participants. Correct answer ratio assigned to “yes” and “no” was 50%. All words were presented in Japanese.

CWST performance was assessed for all participants both within 1 week of the start of the intervention and also after the completion of the intervention. All participants completed a single practice session before each CWST with detailed explanations from the research staff. Reaction time (RT) and error rate (ER) were recorded. Specifically, the (incongruent—neutral) contrast, which is assumed to represent Stroop interference (SI), was calculated to reveal the effect of the 3-month mild-exercise intervention on executive function.

### fNIRS measurements

Cortical hemodynamic changes in the prefrontal cortex during the CWST were monitored with a multichannel fNIRS optical topography system ETG-7000 (Hitachi Medical Corporation, Japan) using two wavelengths of near-infrared light (785 and 830 nm). The device included two sets of 4 × 4 multichannel probe holders consisting of 8 illuminative and 8 detective probes arranged in an alternating fashion at an inter-probe distance of 3 cm, resulting in 24 channels (CH) per set. Probe 5 (between CH 4 and CH 11) in the left probe holder was placed over FT7, with the medial edge of the probe column parallel to the medial line. Similarly, the right probe holder was symmetrically placed on the left hemisphere. Therefore, two fNIRS probes allowed us to cover lateral prefrontal activation foci as in our previous studies [14, 16, 17, 41].

We used a virtual registration method to register fNIRS data to Montreal Neurological Institute (MNI) standard brain space [42–44]. This method enables us to place a virtual probe holder on the scalp to register fNIRS probes and CHs onto reference brains in our MRI database [45, 46] by simulating the holder’s deformation. A statistical analysis of the MNI coordinate values for the fNIRS CHs was performed to obtain the most likely estimate of the location of given CHs for the group of participants and the spatial variability related to the estimation [47]. Lastly, the estimated locations were anatomically labeled using a MATLAB function that reads anatomical labeling information coded in a macro-anatomical brain atlas [48].

### fNIRS data analysis

Optical signals were analyzed based on the modified Beer-Lambert law allowing us to calculate concentration changes of the oxygenated hemoglobin (oxy-Hb) and deoxygenated hemoglobin (deoxy-Hb) in units of millimolar-millimeter (mM・mm) [49, 50]. The sampling rate of optical signals including oxy-Hb and deoxy-Hb was set at 10 Hz. The individual timeline data for the oxy- and deoxy-Hb signal of each CH were preprocessed with a bandpass filter using cut-off frequencies of 0.04 Hz to remove baseline drift and 0.7 Hz to filter out heartbeat pulsations [16, 41]. Channel-wise and participant-wise contrasts were obtained by calculating the inter-trial mean of differences between the oxy- and deoxy-Hb signals of peak (5–12 s after the onset of trial) and baseline (0–3 s before the onset of trial) periods for the older adults. The contrasts obtained were subjected to a second level of random effects group analysis.

As the optical properties of neighboring CHs are known to be similar [51], three or four neighboring CHs were combined to create each region of interest (ROI) based on the LBPA40, which is a widely used method among anatomical labeling systems [48]. In the present study, ROIs included the left dorsolateral PFC (left-DLPFC; CHs 13, 14, 16, and 17), the left ventrolateral PFC (left-VLPFC; CHs 2, 6, and 9), the left frontopolar area (left-FPA; CHs 3, 7, and 10), the right dorsolateral PFC (right-DLFPC; CHs 35, 36, 39, and 40), the right ventrolateral PFC (right-VLPFC; CHs 26, 29, and 33), and the right frontopolar area (right-FPA; CHs 25, 28, and 32). We did not use ROIs as a factor in ANOVA because optical properties in different ROIs can vary systematically, causing bias in statistical analyses. Therefore, we only performed single ROI analyses and used the Bonferroni method for controlling family-wise errors in the current study.

### Neural efficiency

To examine the effects of the intervention on neural efficiency (NE), which relates neurophysiological measures of cortical activity (oxy-Hb changes in ROIs) to an individual’s active cognitive demands (RT for Stroop interference), we calculated NE scores using the following equation [52, 53] and compared changes of the NE of each condition:

$$\mathrm{NE\;score}=\frac{z\left(\mathrm{Stroop\;task\;performance}\right)-z\left(\mathrm{Cortical\;activation}\right)}{\sqrt{2}}$$

where *z* (Stroop task performance) and *z* (Cortical activation) are *z*-scores of RT for SI and SI-related oxy-Hb changes in each ROI, respectively.

### Statistical analyses

Statistical analyses were performed using SPSS Statistical Packages (SPSS 25.0, Chicago, USA). First, baseline characteristics were compared between groups (Con vs ME) with ANOVA for continuous variables and ${x}^{2}$ tests for categorical variables. Then, statistical comparisons were performed using a repeated-measures ANOVA design with the between-subject factor group (Con vs. ME) and the within-subject factor time (baseline vs. after intervention). When a significant *F* value was obtained, a post hoc test using the Bonferroni method was applied. In addition, we compared the differences among the mean values of SI-related variables (e.g., SI_3months_ – SI_baseline)_ between both groups with unpaired *t*-tests to clarify the effect of the 3-month ME intervention.

Next, we examined whether the effect of the mild-exercise intervention on NE differed by age as a subgroup analysis. Within their respective original main groups (Con and ME), we divided participants into two age-related subgroups based on the median age (68 years). Participants below the median age were classified as the younger-aged subgroup (YA; *n* = 44; 23 Con and 21 ME), and participants over the median were classified as the older-aged subgroup (OA; *n* = 37; 18 Con and 19 ME). Then, we performed a repeated measures two-way ANOVA on NE scores for each ROI with group (Con vs. ME) and time (baseline vs. after intervention) for both the YA and OA subgroups followed by Bonferroni’s post hoc test. Finally, we compared the mean difference of NE_3months_ – NE_baseline_ between Con and ME groups for both the YA and the OA subgroups using unpaired *t*-tests. In addition, the Holm method was used to control family-wise error rate.

## Results

### Characteristics of participants and the mild-exercise intervention

There were no significant differences between Con and ME groups in age, sex, height and weight, aerobic fitness level (${\dot{\mathrm V}}_{{\mathrm O}_2\mathrm{peak}}$), years of education, or MMSE score before intervention (see Table 1). Additionally, CWST performance and SI-related oxy-Hb changes at baseline did not differ between the two groups. Participants exercised with an individualized workload (19.2 ± 8.7 watts) corresponding to 35% of their ${\dot{\mathrm V}}_{{\mathrm O}_2\mathrm{peak}}$ for 30 to 50 min (increasing by 10 min per month). Participants’ HR at the beginning of the exercise session increased to 88.8 ± 8.0 beats/min (58.7 ± 5.7% of age-predicted HRmax), and it increased slightly to 92.8 ± 8.6 beats/min (61.4 ± 6.1% of age-predicted HRmax) at the end of the exercise session. Attendance level of the ME intervention program of the forty participants who completed the follow-up assessment was 97.6 ± 3%.

### Aerobic fitness levels

**Fig. 2:**
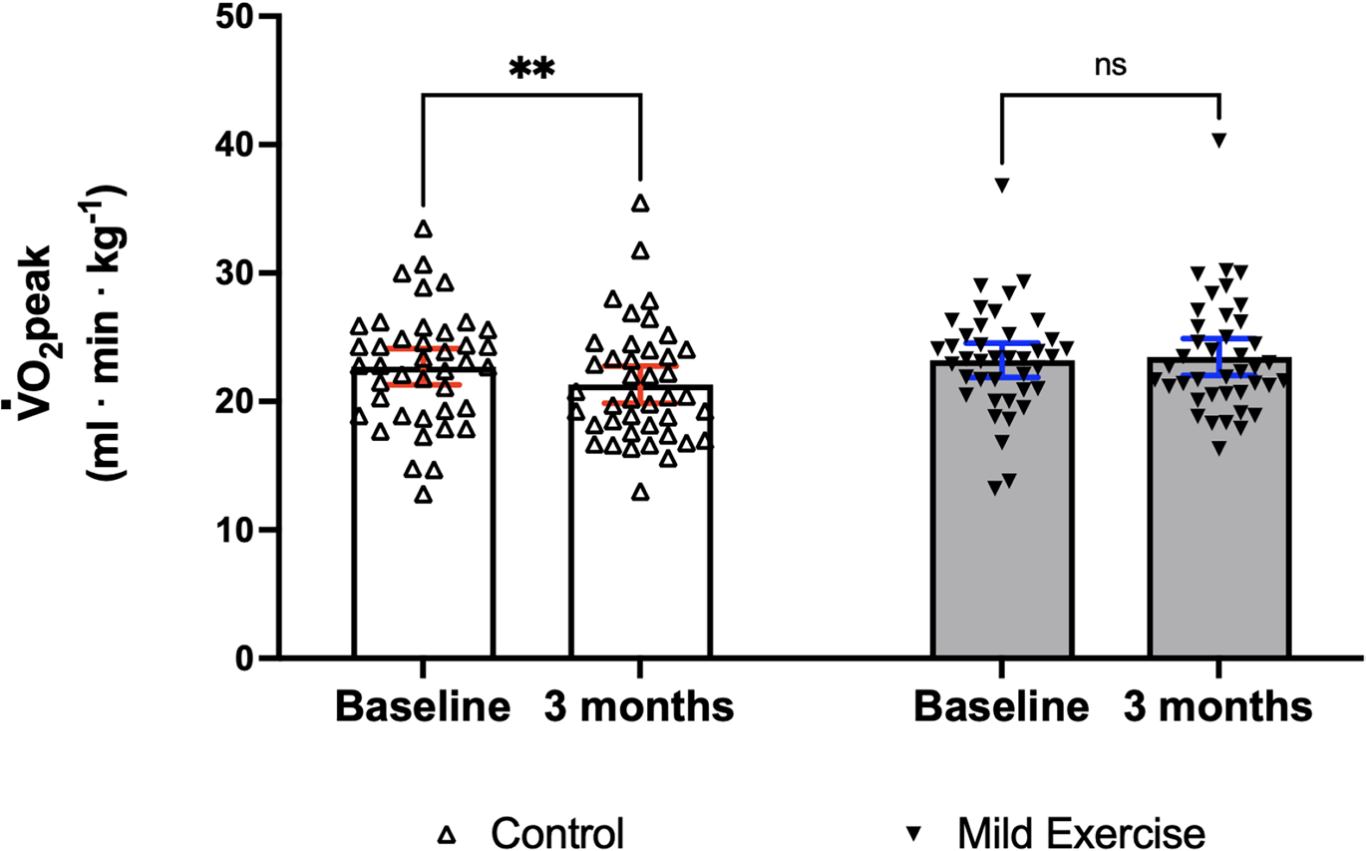
Impact of a 3-month ME intervention on maximal oxygen consumption. Data are mean with 95% CI including individual data. Note. **p < 0.01; ns, not significantly different; ME, mild exercise; CI, confidence interval

Aerobic fitness levels at baseline between Con and ME groups did not significantly differ. There was a significant interaction effect between group (Con/ME) and time (baseline/3 months) on ${\dot{\mathrm V}}_{{\mathrm O}_2\mathrm{peak}}$ (*F* (1,79) = 6.475, *p* = 0.013). A post hoc test using Bonferroni correction demonstrated that there was a significant decrease in ${\dot{\mathrm V}}_{{\mathrm O}_2\mathrm{peak}}$ between baseline and follow-up assessment 3 months later in the Con group (mean of difference: − 1.38, 95% confidence interval [CI] of difference − 2.41 to − 0.34, *p* = 0.006). However, there was no significant change in ${\dot{\mathrm V}}_{{\mathrm O}_2\mathrm{peak}}$ levels between baseline and follow-up assessment 3 months later in the ME group (mean of difference: 0.26, 95% CI of difference − 0.79 to 1.31, *p* > 0.999) (Fig. 2).

### Behavioral outcomes

**Fig. 3:**
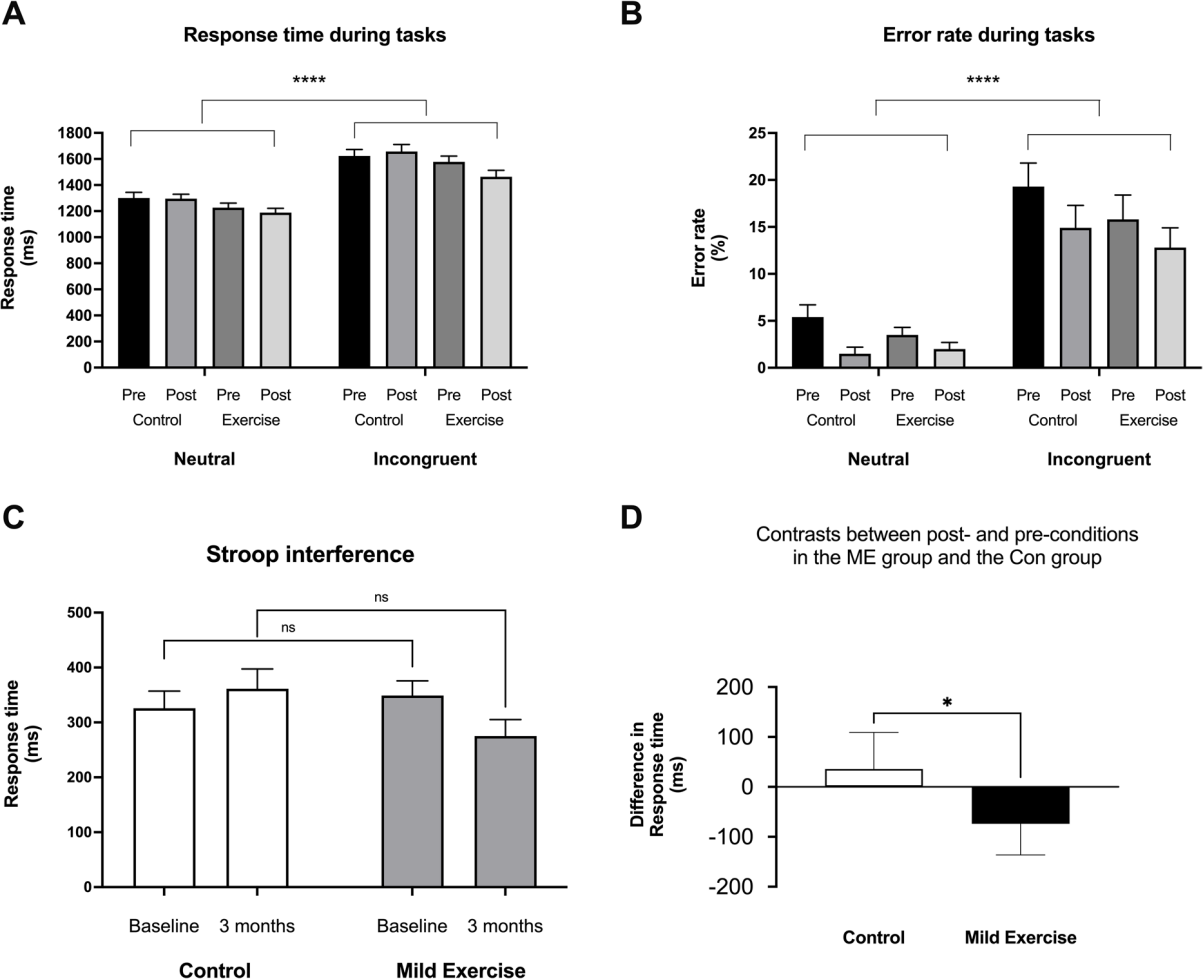
Stroop task performance. Comparison between neutral and incongruent tasks for response time (**A**) and error rate (**B**). The difference in response times between incongruent and neutral tasks indicating Stroop interference for the control and the mild exercise groups is shown in **C**. The contrast between after 3 months and baseline for Stroop interferences is shown for each group (**D**). Data are mean with 95% confidence interval. Note. *p < 0.05; **** *p* < 0.0001; ns, not significantly different

A repeated-measures three-way ANOVA for RT and error rate (ER) with task (neutral/incongruent), group (Con/ME), and time (baseline/3 months) revealed significant main effects of task (*F* (1, 158) = 66.07, *p* < 0.0001, Fig. 3A, and F (1, 158) = 65.33, *p* < 0.0001, Fig. 3B, respectively), which confirmed that Stroop interference (SI) was generally observed between the neutral trials and the incongruent trials (Table 2).

**Table 2:** Stroop task performance

		Control		Mild exercise	
		Baseline	After 3 months	Baseline	After 3 months
Stroop RT	Neutral	1300.5 (1212.5–1388.5)	1295.6 (1226.7–1364.4)	1227.0 (1157.4–1296.7)	1188.4 (1122.5–1254.3)
	Incongruent	1623.8 (1525.0–1722.6)	1657.0 (1548.5–1765.4)	1577.6 (1487.0–1668.2)	1463.7 (1363.2–1564.2)
	Stroop interference	325.7 (262.5–389.0)	361.4 (288.9–434.0)	349.1 (295.3–402.9)	275.2 (215.0–335.5)
Stroop ER	Neutral	5.4 (2.8–7.9)	1.5 (0.1–2.8)	3.5 (1.8–5.2)	2.0 (0.5–3.5)
	Incongruent	19.3 (14.2–24.3)	14.9 (9.9–19.8)	15.8 (10.4–21.1)	12.8 (8.5–17.0)

Response time (ms) and error rate (%) are shown with mean and confidence interval for each condition. Note. *RT* response time, *ER* error rate

The ANOVA for SI-related RT revealed a significant interaction between group and time (*F* (1, 79) = 5.243, *p* = 0.024, Fig. 3C). This interaction is driven by a decreased SI-related RT after the ME intervention (mean difference: − 73.85, 95% CI of difference − 151.6 to 3.9) and an increased SI-related RT in the Con group (mean difference: 35.69, 95% CI of difference − 41.1 to 112.5) as changes in SI-related RTs between the ME group and the Con group were significantly different (t (79) = 2.290, *p* = 0.024, unpaired *t*-test, Fig. 3D). However, there was no significant difference of baseline SI-related RT between the Con group and the ME group (*t* (79) = 0.568, *p* = 0.572, unpaired *t*-test, Fig. 3C). Taken together, these results suggest that the 3-month ME intervention significantly improved executive performance reflected in SI as measured by RT.

### Neural activation in the prefrontal cortex

There were no significant differences between pre-conditions for the ME and Con groups in SI-related oxy-Hb changes (the oxy-Hb contrast between the incongruent condition and neutral condition of the Stroop task) in any ROIs. The ANOVA for oxy-Hb changes in response to SI revealed that there were no significant interactions between group and time factors for all ROIs (Supplementary Fig. 1).

### Exercise-enhanced neural efficiency in the OA group

The ANOVA for neural efficiency score related to SI demonstrated that there were no significant interactions between group and time factors in all ROIs (Supplementary Fig. 2). Comparison and statistical testing of subgroup differences between YA and OA demographic variables, aerobic fitness, and Stroop test performance at baseline are shown in Supplementary Table 1. There were no significant differences between the ME and Con groups of the YA and OA subgroups, respectively.

**Fig. 4:**
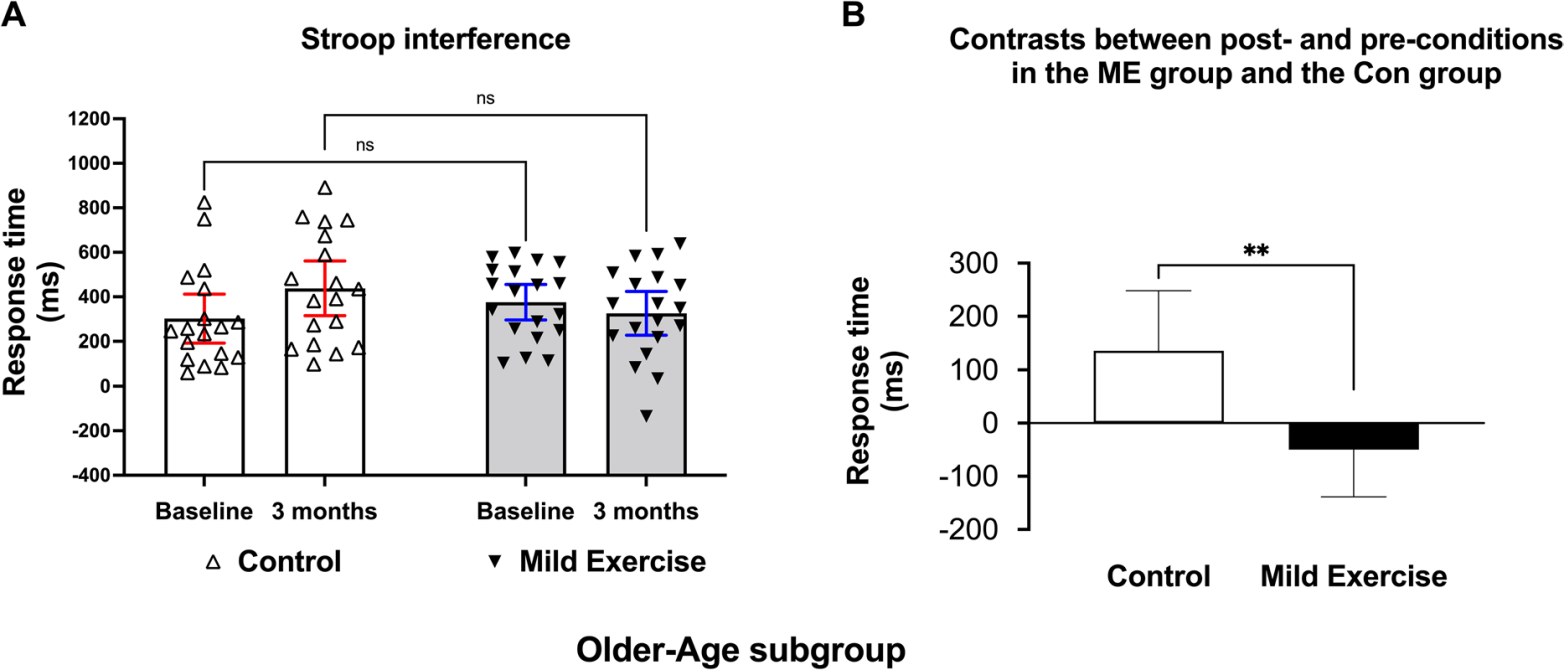
Changes of Stroop performance in the older-age (OA) subgroup. The difference in Stroop interference for the control (Con) and the mild exercise (ME) groups of the OA subgroup including individual data is shown in **A**. The contrast between after 3 months and baseline for Stroop interference in the OA subgroup is shown for Con and ME groups (**B**). Data are mean with 95% confidence interval. Note. **p < 0.01

There was a significant interaction between group and time in SI-related RT in the OA subgroup (*F* (1, 35) = 7.504, *p* = 0.009, Fig. 4A). This interaction may have been driven by a decreased SI after ME intervention (mean difference: − 49.75 ms, 95% CI of difference − 160.4 to 60.9) and increased SI in the Con group (mean difference: 135.80 ms, 95% CI of difference 22.1 to 249.5) as changes in SI-related RTs between the ME group and the Con group were significantly different (*t* (35) = 2.739, *p* = 0.009, unpaired *t-*test, Fig. 4B). However, there were no significant interactions between group and time factors in SI-related RT in the YA subgroup (*F* (1, 42) = 0.706, *p* = 0.406).

**Fig. 5:**
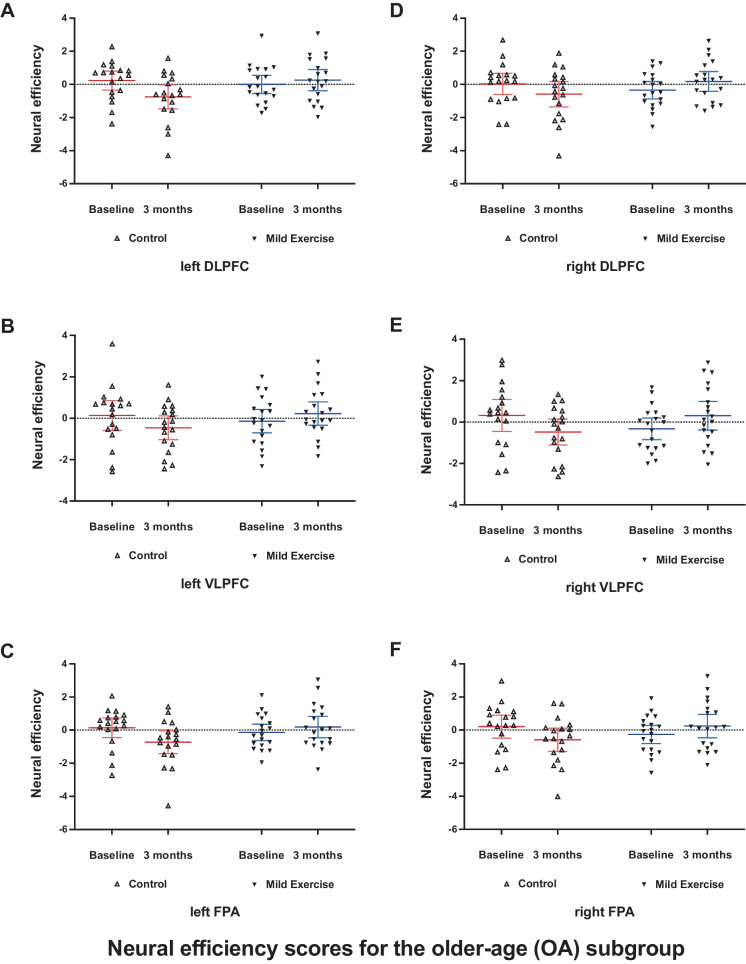
SI-related neural efficiency scores in all ROIs **(**left dorsolateral prefrontal cortex [**A**], left ventrolateral prefrontal cortex [**B**], left frontopolar area [**C**], right dorsolateral prefrontal cortex [**D**], right ventrolateral prefrontal cortex [**E**], right frontopolar area [**F**]) in the older-age (OA) subgroup. Data are mean with 95% confidence interval including individual data

**Fig. 6:**
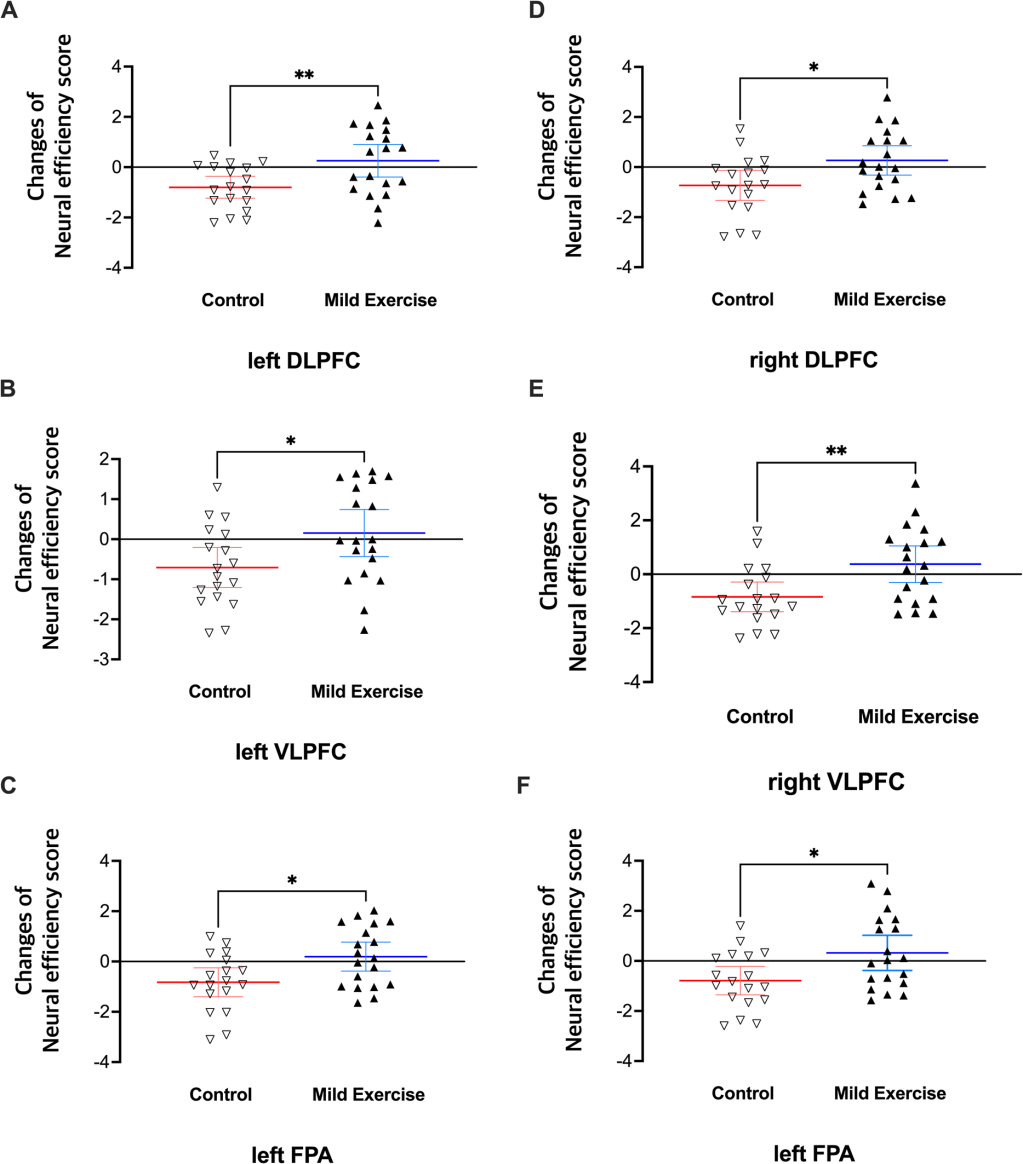
Comparisons of neural efficiency changes in all ROIs **(**left dorsolateral prefrontal cortex [**A**], left ventrolateral prefrontal cortex [**B**], left frontopolar area [**C**], right dorsolateral prefrontal cortex [**D**], right ventrolateral prefrontal cortex [**E**], right frontopolar area [**F**]) between control and mild-exercise in the older-age subgroup. Data are mean with 95% confidence interval including individual data. Note. *p < 0.05, **p < 0.01

The ANOVA performed on the NE score for each of the six ROIs in the OA subgroup revealed significant interactions between group and time factors in all ROIs (left-DLPFC, *F* (1, 35) = 7.352, *p* = 0.010, Fig. 5A; left-VLPFC, *F* (1, 35) = 4.390, *p* = 0.043, Fig. 5B; left-FPA, *F* (1, 35) = 6.293, *p* = 0.017, Fig. 5C; right-DLFPC, *F* (1, 35) = 5.647, *p* = 0.023, Fig. 5D; right-VLPFC, *F* (1, 35) = 8.083, *p* = 0.007, Fig. 5E; right-FPA, *F* (1, 35) = 5.675, *p* = 0.019, Fig. 5F; Holm-corrected). However, there were no significant interactions between group and time factors in all ROIs in the YA subgroup. To examine the interactions in each ROI for the OA subgroup, we compared changes of NE score in the Con and ME groups of the OA subgroup. Changes of NE scores in all ROIs in the ME group were significantly different from those in the Con group (*t* (35) = 2.815, *p* = 0.008, left-DLPFC, Fig. 6A; *t* (35) = 2.332, *p* = 0.026 left-VLPFC, Fig. 6B; *t* (35) = 2.638, *p* = 0.012, left-FPA, Fig. 6C;* t* (35) = 2.508, *p* = 0.017, right-DLPFC, Fig. 6D; *t* (35) = 2.902, *p* = 0.006, right-VLPFC, Fig. 6E; *t* (35) = 2.565, *p* = 0.015, right-FPA, Fig. 6F; Holm-corrected].

## Discussion

Most studies examining the preventive effects of exercise intervention on age-related decline in executive function have focused on cardiovascular exercise protocols such as moderate-intensity exercise. In contrast, we focused on the potential effects of exercise at very light intensity (mild exercise) based on our previous findings that even acute, mild exercise stimulates the DLPFC, which results in better executive performance [14]. Thus, we investigated whether a 3-month ME intervention for older adults would improve executive function and, if so, what neural mechanisms may underlie this effect. Our results indicate that a 3-month ME intervention enhances executive function and related neural efficiency of the prefrontal cortex especially in older adults.

First, we expected that the ME intervention would improve executive performance by shortening SI-related RT mediated by changing task-related cortical activation in the lateral prefrontal cortex as in our previous studies [14, 16, 17]. We observed a significant decrease in SI-related RT in the ME group compared to the Con group after a 3-month mild-exercise intervention. However, we did not observe any significant main effects or interactions between group (Con vs. ME) and time (baseline vs. after intervention) in Oxy-Hb changes in response to the SI in any prefrontal subregions. This suggests that the ME intervention facilitated executive information processing without any increased task-relevant cortical activations in the lateral prefrontal cortex, which was somewhat surprising given our past findings. Then, we tested the hypothesis that neural efficiency, which relates neurophysiological measures of cortical activity to an individual’s cognitive demands, might be increased after the exercise intervention and might be the substrate for enhanced executive performance. However, there were no significant changes of SI-related neural efficiency in the prefrontal cortex. This result is not consistent with previous research that has reported improvement in the efficiency of brain activation during cognitive tasks resulting in improvement of executive function in older adults [8, 26].

Finally, since the impact of a mild-exercise intervention on neural efficiency might differ with age, we divided our participants into two age-dependent subgroups (YA and OA) based on the median split of age in each of their respective groups (ME and Con). We observed that the intervention led to both significantly improved executive performance and significantly improved NE scores in the ME group compared to the Con group, but only for the OA subgroup. These results partially support the notion that ME intervention can stave off age-related executive decline by enhancing NE in the prefrontal cortex in older adults. However, the present findings should be interpreted carefully because our participants included people of various ages, including middle-aged, and we performed a subgroup analysis based on the median age. Therefore, long-term follow-up is necessary to determine if executive decline can be delayed or prevented altogether and whether age is a key moderator for preventing cognitive decline through exercise.

Mild exercise such as walking is considered an appealing exercise prescription among exercise specialists as well as physicians in terms of feasibility and efficacy, especially for sedentary older adults. Acute mild exercise, which is considered a stress-free exercise [54], has been shown to improve executive performance by modulating task-relevant brain activations in the prefrontal cortex [14, 55]. Therefore, it is reasonable to hypothesize that regular mild exercise would be beneficial to the aging brain. It is important to note that the 3-month intervention in this study was completed with a remarkable adherence level (97.6 ± 3% over a total of 36 exercise sessions) and a low drop-out rate (5 of 45 participants dropped out for personal reasons) in the ME group. This means that ME intervention is easily accessible by the older adult population. In addition to practical implications, we have demonstrated a cognitive benefit of the intervention. Executive performance evaluated by SI-related RT was significantly improved after the 3-month mild-exercise intervention in the ME group compared to the Con group, who maintained a sedentary lifestyle. The present results are consistent with previous findings that regular mild-intensity exercise such as walking and calisthenics improve executive function in older adults [13, 56].

Tamura and colleagues (2014) demonstrated that 2 years of ME intervention improved attentional shifting ability by preventing age-related structural changes in prefrontal cortex gray matter. However, the impact of a long-term ME intervention on task-related functional plasticity in the prefrontal cortex remained unknown. To reveal the underlying functional neural substrates of ME-enhanced executive performance, we used an event-related design with fNIRS probes covering the lateral prefrontal cortex during performance of the CWST. In our previous fNIRS studies, we demonstrated that acute exercise, regardless of intensity, increased task-relevant neural activations in the prefrontal cortex in young adults [14, 17, 18]. In addition, acute exercise elicited functional compensatory prefrontal activations in old adults [16]. Acute bouts of exercise may stimulate arousal by increasing the release of various neurotransmitters such as acetylcholine, dopamine, and norepinephrine to task-relevant brain regions, thus making it possible for acute exercise to modulate neural activity temporarily in order to enhance executive performance [14, 57, 58]. However, in the present study, executive performance increased in the ME group compared to the Con group, despite the absence of significant oxy-Hb changes in all ROIs. Thus, we turned our attention to more complex explanations, such as neural efficiency.

It has been previously reported that age-related decline in the efficiency of executive ability may contribute to poor cognitive task performance of older adults [24, 29]. Since recent fNIRS studies have demonstrated that the fNIRS method combined with executive task performance may provide an index of individual neural efficiency [19, 52], we examined whether ME intervention positively modulates NE in the prefrontal cortex. As in previous studies, changes in NE scores with SI in all ROIs significantly decreased with aging in the Con group. However, this age-related NE decline seems to have been blunted in the ME group in the present study. This suggests that there were individual differences among participants that may be associated with the effectiveness of the intervention. To test the hypothesis that the effects of exercise differ by age, we opted to examine the effects of ME on SI-related NE scores in the older adults divided into two age-related subgroups (YA adults vs. OA adults) based on the median age (68 years). Intriguingly, NE scores and executive performance significantly improved only in the OA subgroup of the ME group compared to the Con group. Few studies have reported increased neural efficiency after repeatedly performed cognitive tasks [59, 60]. Others have reported that neural efficiency due to lifelong bilingualism has a positive effect on cognitive control in older adults [61]. Our results are consistent with these studies and further demonstrate that long-term ME intervention is beneficial for improving neural efficiency in older adults over 65 and may ultimately play a role in preventing age-related cognitive decline.

While we have demonstrated that exercise-enhanced executive performance is associated with a form of functional neuroplasticity after a long-term ME intervention in older adults, it remains unknown whether and how such an exercise intervention may maintain executive brain function in the aging brain. Work in animal models has suggested that exercise-induced neurotrophins, such as brain-derived neurotrophic factor (BDNF), insulin-like growth factor I (IGF-I), and nerve growth factor (NGF), are involved in brain plasticity by increasing neuronal survival and maintenance in the face of aging [62–65]. In addition, exercise-increased density of dendritic spines and numbers of synapses in the medial prefrontal cortex as well as the hippocampus are putative neural substrates for better cognitive performance [66, 67]. In addition to exercise-induced neurobiological factors, brain functional connectivity might be modulated with regular exercise, including activities such as a Tai-chi program, in both younger and older adults [68, 69]. To bridge human studies with more mechanistic studies in animals, future studies in humans could combine cutting-edge neuroimaging methods, such as high-resolution diffusion imaging and high-resolution resting state functional magnetic resonance imaging, which can assess regional changes in both structure and function with greater precision.

## Conclusion

In the present study, we revealed that a long-term mild-exercise intervention improves executive performance in middle-aged and elderly adults. However, there were no significant increases in task-related neural activation or task-related neural efficiency in the prefrontal cortex. Interestingly, it was revealed that a 3-month mild-exercise intervention improves executive function by modulating the neural efficiency of the prefrontal cortex, especially in older adults, via a subgroup analysis. Our results also demonstrate the practicality and high accessibility of a mild-exercise regimen in older adults, which has significant public health implications. Future work with longer-term interventions will be equipped to determine whether such a mild exercise regimen can protect the aging brain from cognitive decline.

### Supplementary Information

Below is the link to the electronic supplementary material.

Supplementary file1 (DOCX 239 KB)

## Data Availability

The data that support the findings of this study are available from the corresponding author, HS, upon reasonable request.
