# Supplementary material for: Mild exercise improves executive function with increasing neural efficiency in the prefrontal cortex of older adults
Source: GeroScience. 2023 Jun 15;46(1):309–25. doi: 10.1007/s11357-023-00816-3 (PMC10828372; doi:10.1007/s11357-023-00816-3)
Supplement: Supplementary file 1 — Supplementary file1 (DOCX 239 KB) [file 11357_2023_816_MOESM1_ESM.docx]

**Supp. Fig. 1.** SI-related oxy-Hb changes in all ROIs [left dorsolateral prefrontal cortex (A), left ventrolateral prefrontal cortex (B), left frontopolar area (C), right dorsolateral prefrontal cortex (D), right ventrolateral prefrontal cortex (E), right frontopolar area (F)]. Data are mean with 95% confidence interval including an individual data.

Supp. Fig. 2. SI-related neural efficiency scores in all ROIs (left dorsolateral prefrontal cortex (A), left ventrolateral prefrontal cortex (B), left frontopolar area (C), right dorsolateral prefrontal cortex (D), right ventrolateral prefrontal cortex (E), right frontopolar area (F)). Data are mean with 95% confidence interval including an individual data.

**Supp. Table 1.** Participants’ baseline characteristics in the YA and OA subgroups

|  | | Younger-age  n = 44, 7 males | | Older-age  n = 44, 7 males | |
| --- | --- | --- | --- | --- | --- |
|  |  | Mild Exercise  n = 21, 5 males | Control  n = 23, 2 males | Mild Exercise  n = 19, 5 males | Control  n = 18, 8 males |
| Age  (years) | | 64.8  (63.3 – 66.2) | 63.5  (61.9 – 65.1) | 72.9  (71.6 – 74.3) | 73.2  (72.1 – 74.2) |
| Education  (years) | | 13.9  (12.7 – 15.0) | 13.8  (13.0 – 14.6) | 12.9  (12.1 – 13.8) | 13.9  (12.9 – 14.9) |
| MMSE  (score) | | 29.4  (28.9 – 29.8) | 29.4  (29.0 – 29.9) | 28.7  (28.2 – 29.3) | 28.4  (27.7 – 29.2) |
| VO_2peak_  (ml・min・kg^-1^) | | 24.4  (22.4 – 26.4) | 23.7  (22.1 – 25.4) | 21.9  (20.1 – 23.6) | 21.4  (18.9 – 23.8) |
| Stroop  RT | Neutral | 1170.8  (1075.1 – 1266.5) | 1250.5  (1148.1 – 1352.9) | 1289.2  (1186.0 – 1392.4) | 1364.3  (1205.1 – 1523.6) |
|  | Incongruent | 1507.4  (1378.3 – 1636.5) | 1582.8  (1450.5 – 1715.1) | 1655.2  (1525.5 – 1785.0) | 1676.2  (1515.7 – 1836.6) |
|  | Interference | 324.9  (246.9 – 402.9) | 344.1  (264.1 – 424.2) | 375.9  (296.2 – 455.5) | 302.2  (192.1 – 412.3) |

Participants’ baseline characteristics and response time (ms) for the Stroop task of YA and OA group are shown with mean and confidence interval for each condition. Note: *YO,* younger-age; *OA,* older-age; *MMSE,* Mini-Mental Status Examination; *VO_2peak_*, peak oxygen consumption; *RT* response time.
